# Supplementary material for: Pyruvate Dehydrogenase Kinase 1 inhibition mediated oxidative phosphorylation enhancement in cartilage promotes osteoarthritis progression
Source: BMC Musculoskelet Disord. 2023 Jul 20;24:597. doi: 10.1186/s12891-023-06585-6 (PMC10357736; doi:10.1186/s12891-023-06585-6)
Supplement: Supplementary file 1 — Additional file 1. [file 12891_2023_6585_MOESM1_ESM.zip › KEGG.Gsea.1653622432464/KEGG_INSULIN_SIGNALING_PATHWAY.html]

Details for gene set KEGG\_INSULIN\_SIGNALING\_PATHWAY[GSEA]

|  || Dataset | 55457\_collapsed\_to\_symbols.cls.cls #OA\_versus\_normal.cls.cls #OA\_versus\_normal\_repos |
| Phenotype | cls.cls#OA\_versus\_normal\_repos |
| Upregulated in class | normal |
| GeneSet | KEGG\_INSULIN\_SIGNALING\_PATHWAY |
| Enrichment Score (ES) | -0.31765807 |
| Normalized Enrichment Score (NES) | -1.4322721 |
| Nominal p-value | 0.010574019 |
| FDR q-value | 0.21899728 |
| FWER p-Value | 0.986 |
Table: GSEA Results Summary

  

Fig 1: Enrichment plot: KEGG\_INSULIN\_SIGNALING\_PATHWAY      
 Profile of the Running ES Score & Positions of GeneSet Members on the Rank Ordered List

  

| SYMBOL | TITLE | RANK IN GENE LIST | RANK METRIC SCORE | RUNNING ES | CORE ENRICHMENT || 1 | PPP1R3C | protein phosphatase 1 regulatory subunit 3C [Source:HGNC Symbol;Acc:HGNC:9293] | 41 | 1.008 | 0.0190 | No |
| 2 | PRKAG1 | protein kinase AMP-activated non-catalytic subunit gamma 1 [Source:HGNC Symbol;Acc:HGNC:9385] | 138 | 0.870 | 0.0306 | No |
| 3 | FLOT1 | flotillin 1 [Source:HGNC Symbol;Acc:HGNC:3757] | 409 | 0.703 | 0.0250 | No |
| 4 | PPP1CA | protein phosphatase 1 catalytic subunit alpha [Source:HGNC Symbol;Acc:HGNC:9281] | 603 | 0.622 | 0.0236 | No |
| 5 | PPP1R3D | protein phosphatase 1 regulatory subunit 3D [Source:HGNC Symbol;Acc:HGNC:9294] | 708 | 0.591 | 0.0284 | No |
| 6 | MAPK9 | mitogen-activated protein kinase 9 [Source:HGNC Symbol;Acc:HGNC:6886] | 721 | 0.588 | 0.0404 | No |
| 7 | PPP1CC | protein phosphatase 1 catalytic subunit gamma [Source:HGNC Symbol;Acc:HGNC:9283] | 744 | 0.583 | 0.0515 | No |
| 8 | MAPK3 | mitogen-activated protein kinase 3 [Source:HGNC Symbol;Acc:HGNC:6877] | 757 | 0.579 | 0.0633 | No |
| 9 | PCK2 | "phosphoenolpyruvate carboxykinase 2, mitochondrial [Source:HGNC Symbol;Acc:HGNC:8725]" | 759 | 0.579 | 0.0760 | No |
| 10 | PRKACB | protein kinase cAMP-activated catalytic subunit beta [Source:HGNC Symbol;Acc:HGNC:9381] | 771 | 0.576 | 0.0878 | No |
| 11 | FBP1 | fructose-bisphosphatase 1 [Source:HGNC Symbol;Acc:HGNC:3606] | 772 | 0.576 | 0.1005 | No |
| 12 | PHKB | phosphorylase kinase regulatory subunit beta [Source:HGNC Symbol;Acc:HGNC:8927] | 853 | 0.554 | 0.1064 | No |
| 13 | CALM3 | calmodulin 3 [Source:HGNC Symbol;Acc:HGNC:1449] | 1039 | 0.510 | 0.1032 | No |
| 14 | AKT1 | AKT serine/threonine kinase 1 [Source:HGNC Symbol;Acc:HGNC:391] | 1192 | 0.478 | 0.1018 | No |
| 15 | CALML5 | calmodulin like 5 [Source:HGNC Symbol;Acc:HGNC:18180] | 1261 | 0.465 | 0.1067 | No |
| 16 | EIF4E2 | eukaryotic translation initiation factor 4E family member 2 [Source:HGNC Symbol;Acc:HGNC:3293] | 1301 | 0.458 | 0.1137 | No |
| 17 | PYGM | "glycogen phosphorylase, muscle associated [Source:HGNC Symbol;Acc:HGNC:9726]" | 1952 | 0.367 | 0.0709 | No |
| 18 | CRK | "CRK proto-oncogene, adaptor protein [Source:HGNC Symbol;Acc:HGNC:2362]" | 2018 | 0.360 | 0.0737 | No |
| 19 | PIK3CB | "phosphatidylinositol-4,5-bisphosphate 3-kinase catalytic subunit beta [Source:HGNC Symbol;Acc:HGNC:8976]" | 2186 | 0.340 | 0.0681 | No |
| 20 | CRKL | "CRK like proto-oncogene, adaptor protein [Source:HGNC Symbol;Acc:HGNC:2363]" | 2283 | 0.329 | 0.0679 | No |
| 21 | PYGB | glycogen phosphorylase B [Source:HGNC Symbol;Acc:HGNC:9723] | 2400 | 0.315 | 0.0657 | No |
| 22 | SHC1 | SHC adaptor protein 1 [Source:HGNC Symbol;Acc:HGNC:10840] | 2485 | 0.304 | 0.0658 | No |
| 23 | PPP1R3A | protein phosphatase 1 regulatory subunit 3A [Source:HGNC Symbol;Acc:HGNC:9291] | 2732 | 0.277 | 0.0527 | No |
| 24 | PRKCZ | protein kinase C zeta [Source:HGNC Symbol;Acc:HGNC:9412] | 2760 | 0.274 | 0.0566 | No |
| 25 | EXOC7 | exocyst complex component 7 [Source:HGNC Symbol;Acc:HGNC:23214] | 2973 | 0.253 | 0.0456 | No |
| 26 | INPP5D | inositol polyphosphate-5-phosphatase D [Source:HGNC Symbol;Acc:HGNC:6079] | 3085 | 0.241 | 0.0422 | No |
| 27 | ELK1 | ETS transcription factor ELK1 [Source:HGNC Symbol;Acc:HGNC:3321] | 3357 | 0.216 | 0.0257 | No |
| 28 | FBP2 | fructose-bisphosphatase 2 [Source:HGNC Symbol;Acc:HGNC:3607] | 3763 | 0.180 | -0.0020 | No |
| 29 | BAD | BCL2 associated agonist of cell death [Source:HGNC Symbol;Acc:HGNC:936] | 3788 | 0.178 | 0.0000 | No |
| 30 | HK3 | hexokinase 3 [Source:HGNC Symbol;Acc:HGNC:4925] | 3792 | 0.178 | 0.0037 | No |
| 31 | PYGL | glycogen phosphorylase L [Source:HGNC Symbol;Acc:HGNC:9725] | 3826 | 0.174 | 0.0049 | No |
| 32 | INS | insulin [Source:HGNC Symbol;Acc:HGNC:6081] | 3885 | 0.166 | 0.0040 | No |
| 33 | PRKAG2 | protein kinase AMP-activated non-catalytic subunit gamma 2 [Source:HGNC Symbol;Acc:HGNC:9386] | 4362 | 0.123 | -0.0305 | No |
| 34 | SREBF1 | sterol regulatory element binding transcription factor 1 [Source:HGNC Symbol;Acc:HGNC:11289] | 4729 | 0.093 | -0.0571 | No |
| 35 | PTPRF | protein tyrosine phosphatase receptor type F [Source:HGNC Symbol;Acc:HGNC:9670] | 4839 | 0.084 | -0.0638 | No |
| 36 | G6PC1 | glucose-6-phosphatase catalytic subunit 1 [Source:HGNC Symbol;Acc:HGNC:4056] | 5389 | 0.041 | -0.1059 | No |
| 37 | RPS6KB2 | ribosomal protein S6 kinase B2 [Source:HGNC Symbol;Acc:HGNC:10437] | 5496 | 0.033 | -0.1135 | No |
| 38 | GRB2 | growth factor receptor bound protein 2 [Source:HGNC Symbol;Acc:HGNC:4566] | 5697 | 0.016 | -0.1288 | No |
| 39 | SHC2 | SHC adaptor protein 2 [Source:HGNC Symbol;Acc:HGNC:29869] | 5738 | 0.012 | -0.1316 | No |
| 40 | PRKAR1B | protein kinase cAMP-dependent type I regulatory subunit beta [Source:HGNC Symbol;Acc:HGNC:9390] | 5806 | 0.008 | -0.1367 | No |
| 41 | MKNK1 | MAPK interacting serine/threonine kinase 1 [Source:HGNC Symbol;Acc:HGNC:7110] | 5830 | 0.007 | -0.1384 | No |
| 42 | EIF4EBP1 | eukaryotic translation initiation factor 4E binding protein 1 [Source:HGNC Symbol;Acc:HGNC:3288] | 5871 | 0.004 | -0.1414 | No |
| 43 | IKBKB | inhibitor of nuclear factor kappa B kinase subunit beta [Source:HGNC Symbol;Acc:HGNC:5960] | 5914 | 0.001 | -0.1447 | No |
| 44 | PDE3B | phosphodiesterase 3B [Source:HGNC Symbol;Acc:HGNC:8779] | 6077 | -0.010 | -0.1571 | No |
| 45 | IRS4 | insulin receptor substrate 4 [Source:HGNC Symbol;Acc:HGNC:6128] | 6106 | -0.013 | -0.1590 | No |
| 46 | RAF1 | "Raf-1 proto-oncogene, serine/threonine kinase [Source:HGNC Symbol;Acc:HGNC:9829]" | 6187 | -0.019 | -0.1649 | No |
| 47 | GYS2 | glycogen synthase 2 [Source:HGNC Symbol;Acc:HGNC:4707] | 6316 | -0.029 | -0.1742 | No |
| 48 | CALML3 | calmodulin like 3 [Source:HGNC Symbol;Acc:HGNC:1452] | 6348 | -0.032 | -0.1760 | No |
| 49 | PHKG1 | phosphorylase kinase catalytic subunit gamma 1 [Source:HGNC Symbol;Acc:HGNC:8930] | 6451 | -0.039 | -0.1831 | No |
| 50 | FOXO1 | forkhead box O1 [Source:HGNC Symbol;Acc:HGNC:3819] | 6511 | -0.043 | -0.1868 | No |
| 51 | HK1 | hexokinase 1 [Source:HGNC Symbol;Acc:HGNC:4922] | 6728 | -0.058 | -0.2024 | No |
| 52 | MAPK1 | mitogen-activated protein kinase 1 [Source:HGNC Symbol;Acc:HGNC:6871] | 6853 | -0.068 | -0.2106 | No |
| 53 | TSC2 | TSC complex subunit 2 [Source:HGNC Symbol;Acc:HGNC:12363] | 6946 | -0.075 | -0.2162 | No |
| 54 | PRKAR2B | protein kinase cAMP-dependent type II regulatory subunit beta [Source:HGNC Symbol;Acc:HGNC:9392] | 7034 | -0.082 | -0.2212 | No |
| 55 | LIPE | "lipase E, hormone sensitive type [Source:HGNC Symbol;Acc:HGNC:6621]" | 7185 | -0.095 | -0.2308 | No |
| 56 | NRAS | "NRAS proto-oncogene, GTPase [Source:HGNC Symbol;Acc:HGNC:7989]" | 7300 | -0.102 | -0.2375 | No |
| 57 | GYS1 | glycogen synthase 1 [Source:HGNC Symbol;Acc:HGNC:4706] | 7345 | -0.105 | -0.2386 | No |
| 58 | PIK3CD | "phosphatidylinositol-4,5-bisphosphate 3-kinase catalytic subunit delta [Source:HGNC Symbol;Acc:HGNC:8977]" | 7405 | -0.111 | -0.2408 | No |
| 59 | PIK3CA | "phosphatidylinositol-4,5-bisphosphate 3-kinase catalytic subunit alpha [Source:HGNC Symbol;Acc:HGNC:8975]" | 7733 | -0.136 | -0.2634 | No |
| 60 | PHKA1 | phosphorylase kinase regulatory subunit alpha 1 [Source:HGNC Symbol;Acc:HGNC:8925] | 7896 | -0.147 | -0.2729 | No |
| 61 | PIK3CG | "phosphatidylinositol-4,5-bisphosphate 3-kinase catalytic subunit gamma [Source:HGNC Symbol;Acc:HGNC:8978]" | 7953 | -0.152 | -0.2739 | No |
| 62 | HRAS | "HRas proto-oncogene, GTPase [Source:HGNC Symbol;Acc:HGNC:5173]" | 7977 | -0.153 | -0.2723 | No |
| 63 | AKT3 | AKT serine/threonine kinase 3 [Source:HGNC Symbol;Acc:HGNC:393] | 8008 | -0.156 | -0.2713 | No |
| 64 | SOS2 | SOS Ras/Rho guanine nucleotide exchange factor 2 [Source:HGNC Symbol;Acc:HGNC:11188] | 8058 | -0.160 | -0.2716 | No |
| 65 | ARAF | "A-Raf proto-oncogene, serine/threonine kinase [Source:HGNC Symbol;Acc:HGNC:646]" | 8092 | -0.163 | -0.2706 | No |
| 66 | INPP5K | inositol polyphosphate-5-phosphatase K [Source:HGNC Symbol;Acc:HGNC:33882] | 8103 | -0.163 | -0.2678 | No |
| 67 | BRAF | "B-Raf proto-oncogene, serine/threonine kinase [Source:HGNC Symbol;Acc:HGNC:1097]" | 8171 | -0.169 | -0.2693 | No |
| 68 | PHKA2 | phosphorylase kinase regulatory subunit alpha 2 [Source:HGNC Symbol;Acc:HGNC:8926] | 8333 | -0.181 | -0.2779 | No |
| 69 | SORBS1 | sorbin and SH3 domain containing 1 [Source:HGNC Symbol;Acc:HGNC:14565] | 8509 | -0.194 | -0.2873 | No |
| 70 | PRKAB1 | protein kinase AMP-activated non-catalytic subunit beta 1 [Source:HGNC Symbol;Acc:HGNC:9378] | 8708 | -0.210 | -0.2982 | No |
| 71 | PIK3R3 | phosphoinositide-3-kinase regulatory subunit 3 [Source:HGNC Symbol;Acc:HGNC:8981] | 8801 | -0.217 | -0.3007 | No |
| 72 | PIK3R5 | phosphoinositide-3-kinase regulatory subunit 5 [Source:HGNC Symbol;Acc:HGNC:30035] | 8804 | -0.217 | -0.2960 | No |
| 73 | PTPN1 | protein tyrosine phosphatase non-receptor type 1 [Source:HGNC Symbol;Acc:HGNC:9642] | 8829 | -0.220 | -0.2931 | No |
| 74 | PRKAA2 | protein kinase AMP-activated catalytic subunit alpha 2 [Source:HGNC Symbol;Acc:HGNC:9377] | 8864 | -0.222 | -0.2909 | No |
| 75 | MAPK8 | mitogen-activated protein kinase 8 [Source:HGNC Symbol;Acc:HGNC:6881] | 8956 | -0.228 | -0.2930 | No |
| 76 | G6PC2 | glucose-6-phosphatase catalytic subunit 2 [Source:HGNC Symbol;Acc:HGNC:28906] | 9088 | -0.239 | -0.2980 | No |
| 77 | PCK1 | phosphoenolpyruvate carboxykinase 1 [Source:HGNC Symbol;Acc:HGNC:8724] | 9100 | -0.239 | -0.2936 | No |
| 78 | ACACA | acetyl-CoA carboxylase alpha [Source:HGNC Symbol;Acc:HGNC:84] | 9177 | -0.246 | -0.2941 | No |
| 79 | CBL | Cbl proto-oncogene [Source:HGNC Symbol;Acc:HGNC:1541] | 9479 | -0.272 | -0.3117 | Yes |
| 80 | CBLC | Cbl proto-oncogene C [Source:HGNC Symbol;Acc:HGNC:15961] | 9553 | -0.279 | -0.3112 | Yes |
| 81 | SHC3 | SHC adaptor protein 3 [Source:HGNC Symbol;Acc:HGNC:18181] | 9608 | -0.284 | -0.3092 | Yes |
| 82 | FLOT2 | flotillin 2 [Source:HGNC Symbol;Acc:HGNC:3758] | 9680 | -0.290 | -0.3084 | Yes |
| 83 | MAPK10 | mitogen-activated protein kinase 10 [Source:HGNC Symbol;Acc:HGNC:6872] | 9699 | -0.291 | -0.3034 | Yes |
| 84 | EIF4E | eukaryotic translation initiation factor 4E [Source:HGNC Symbol;Acc:HGNC:3287] | 9709 | -0.293 | -0.2977 | Yes |
| 85 | SOCS2 | suppressor of cytokine signaling 2 [Source:HGNC Symbol;Acc:HGNC:19382] | 9802 | -0.302 | -0.2982 | Yes |
| 86 | MAP2K2 | mitogen-activated protein kinase kinase 2 [Source:HGNC Symbol;Acc:HGNC:6842] | 9932 | -0.315 | -0.3014 | Yes |
| 87 | HK2 | hexokinase 2 [Source:HGNC Symbol;Acc:HGNC:4923] | 10030 | -0.325 | -0.3018 | Yes |
| 88 | PRKAR1A | protein kinase cAMP-dependent type I regulatory subunit alpha [Source:HGNC Symbol;Acc:HGNC:9388] | 10093 | -0.330 | -0.2994 | Yes |
| 89 | PKLR | pyruvate kinase L/R [Source:HGNC Symbol;Acc:HGNC:9020] | 10120 | -0.333 | -0.2941 | Yes |
| 90 | CALM1 | calmodulin 1 [Source:HGNC Symbol;Acc:HGNC:1442] | 10136 | -0.334 | -0.2879 | Yes |
| 91 | GCK | glucokinase [Source:HGNC Symbol;Acc:HGNC:4195] | 10137 | -0.335 | -0.2806 | Yes |
| 92 | MAP2K1 | mitogen-activated protein kinase kinase 1 [Source:HGNC Symbol;Acc:HGNC:6840] | 10197 | -0.340 | -0.2777 | Yes |
| 93 | RPS6KB1 | ribosomal protein S6 kinase B1 [Source:HGNC Symbol;Acc:HGNC:10436] | 10224 | -0.343 | -0.2722 | Yes |
| 94 | PRKACG | protein kinase cAMP-activated catalytic subunit gamma [Source:HGNC Symbol;Acc:HGNC:9382] | 10466 | -0.370 | -0.2829 | Yes |
| 95 | PPARGC1A | PPARG coactivator 1 alpha [Source:HGNC Symbol;Acc:HGNC:9237] | 10504 | -0.374 | -0.2776 | Yes |
| 96 | KRAS | "KRAS proto-oncogene, GTPase [Source:HGNC Symbol;Acc:HGNC:6407]" | 10544 | -0.377 | -0.2723 | Yes |
| 97 | CALM2 | calmodulin 2 [Source:HGNC Symbol;Acc:HGNC:1445] | 10579 | -0.382 | -0.2666 | Yes |
| 98 | PRKACA | protein kinase cAMP-activated catalytic subunit alpha [Source:HGNC Symbol;Acc:HGNC:9380] | 10611 | -0.384 | -0.2605 | Yes |
| 99 | PRKAR2A | protein kinase cAMP-dependent type II regulatory subunit alpha [Source:HGNC Symbol;Acc:HGNC:9391] | 10664 | -0.389 | -0.2560 | Yes |
| 100 | SLC2A4 | solute carrier family 2 member 4 [Source:HGNC Symbol;Acc:HGNC:11009] | 10764 | -0.400 | -0.2550 | Yes |
| 101 | PHKG2 | phosphorylase kinase catalytic subunit gamma 2 [Source:HGNC Symbol;Acc:HGNC:8931] | 10830 | -0.408 | -0.2511 | Yes |
| 102 | ACACB | acetyl-CoA carboxylase beta [Source:HGNC Symbol;Acc:HGNC:85] | 10955 | -0.425 | -0.2514 | Yes |
| 103 | PIK3R2 | phosphoinositide-3-kinase regulatory subunit 2 [Source:HGNC Symbol;Acc:HGNC:8980] | 10956 | -0.425 | -0.2421 | Yes |
| 104 | PIK3R1 | phosphoinositide-3-kinase regulatory subunit 1 [Source:HGNC Symbol;Acc:HGNC:8979] | 11099 | -0.443 | -0.2434 | Yes |
| 105 | GSK3B | glycogen synthase kinase 3 beta [Source:HGNC Symbol;Acc:HGNC:4617] | 11157 | -0.451 | -0.2380 | Yes |
| 106 | IRS2 | insulin receptor substrate 2 [Source:HGNC Symbol;Acc:HGNC:6126] | 11390 | -0.485 | -0.2455 | Yes |
| 107 | PRKAA1 | protein kinase AMP-activated catalytic subunit alpha 1 [Source:HGNC Symbol;Acc:HGNC:9376] | 11499 | -0.501 | -0.2429 | Yes |
| 108 | PDE3A | phosphodiesterase 3A [Source:HGNC Symbol;Acc:HGNC:8778] | 11546 | -0.507 | -0.2353 | Yes |
| 109 | TSC1 | TSC complex subunit 1 [Source:HGNC Symbol;Acc:HGNC:12362] | 11575 | -0.511 | -0.2263 | Yes |
| 110 | PRKX | protein kinase X-linked [Source:HGNC Symbol;Acc:HGNC:9441] | 11634 | -0.522 | -0.2193 | Yes |
| 111 | RPS6 | ribosomal protein S6 [Source:HGNC Symbol;Acc:HGNC:10429] | 11662 | -0.526 | -0.2098 | Yes |
| 112 | TRIP10 | thyroid hormone receptor interactor 10 [Source:HGNC Symbol;Acc:HGNC:12304] | 11710 | -0.535 | -0.2017 | Yes |
| 113 | PPP1CB | protein phosphatase 1 catalytic subunit beta [Source:HGNC Symbol;Acc:HGNC:9282] | 11737 | -0.539 | -0.1919 | Yes |
| 114 | PDPK1 | 3-phosphoinositide dependent protein kinase 1 [Source:HGNC Symbol;Acc:HGNC:8816] | 11839 | -0.557 | -0.1875 | Yes |
| 115 | IRS1 | insulin receptor substrate 1 [Source:HGNC Symbol;Acc:HGNC:6125] | 11841 | -0.557 | -0.1753 | Yes |
| 116 | SOS1 | SOS Ras/Rac guanine nucleotide exchange factor 1 [Source:HGNC Symbol;Acc:HGNC:11187] | 12041 | -0.603 | -0.1776 | Yes |
| 117 | MTOR | mechanistic target of rapamycin kinase [Source:HGNC Symbol;Acc:HGNC:3942] | 12047 | -0.605 | -0.1647 | Yes |
| 118 | RHOQ | ras homolog family member Q [Source:HGNC Symbol;Acc:HGNC:17736] | 12053 | -0.607 | -0.1517 | Yes |
| 119 | FASN | fatty acid synthase [Source:HGNC Symbol;Acc:HGNC:3594] | 12194 | -0.643 | -0.1485 | Yes |
| 120 | MKNK2 | MAPK interacting serine/threonine kinase 2 [Source:HGNC Symbol;Acc:HGNC:7111] | 12236 | -0.655 | -0.1373 | Yes |
| 121 | PRKCI | protein kinase C iota [Source:HGNC Symbol;Acc:HGNC:9404] | 12444 | -0.723 | -0.1376 | Yes |
| 122 | SOCS3 | suppressor of cytokine signaling 3 [Source:HGNC Symbol;Acc:HGNC:19391] | 12453 | -0.725 | -0.1223 | Yes |
| 123 | SOCS1 | suppressor of cytokine signaling 1 [Source:HGNC Symbol;Acc:HGNC:19383] | 12664 | -0.836 | -0.1203 | Yes |
| 124 | CBLB | Cbl proto-oncogene B [Source:HGNC Symbol;Acc:HGNC:1542] | 12725 | -0.887 | -0.1055 | Yes |
| 125 | PRKAB2 | protein kinase AMP-activated non-catalytic subunit beta 2 [Source:HGNC Symbol;Acc:HGNC:9379] | 12731 | -0.893 | -0.0862 | Yes |
| 126 | SH2B2 | SH2B adaptor protein 2 [Source:HGNC Symbol;Acc:HGNC:17381] | 12769 | -0.930 | -0.0686 | Yes |
| 127 | INSR | insulin receptor [Source:HGNC Symbol;Acc:HGNC:6091] | 12856 | -1.071 | -0.0518 | Yes |
| 128 | RAPGEF1 | Rap guanine nucleotide exchange factor 1 [Source:HGNC Symbol;Acc:HGNC:4568] | 12860 | -1.098 | -0.0278 | Yes |
| 129 | AKT2 | AKT serine/threonine kinase 2 [Source:HGNC Symbol;Acc:HGNC:392] | 12896 | -1.404 | 0.0003 | Yes |
Table: GSEA details [plain text format]

  

Fig 2: KEGG\_INSULIN\_SIGNALING\_PATHWAY      
 Blue-Pink O' Gram in the Space of the Analyzed GeneSet

  

Fig 3: KEGG\_INSULIN\_SIGNALING\_PATHWAY: Random ES distribution      
 Gene set null distribution of ES for **KEGG\_INSULIN\_SIGNALING\_PATHWAY**
